# Parallelization of multicategory support vector machines (PMC-SVM) for classifying microarray data

**DOI:** 10.1186/1471-2105-7-S4-S15

**Published:** 2006-12-12

**Authors:** Chaoyang Zhang, Peng Li, Arun Rajendran, Youping Deng, Dequan Chen

**Affiliations:** 1 School of Computing, University of Southern Mississippi, Hattiesburg, MS 39406, USA; 2 Department of Biological Sciences, University of Southern Mississippi, Hattiesburg, MS 39406, USA; 3 Dequan Chen, Institute for Retina Research, Dallas, TX 75231, USA

## Abstract

**Background:**

Multicategory Support Vector Machines (MC-SVM) are powerful classification systems with excellent performance in a variety of data classification problems. Since the process of generating models in traditional multicategory support vector machines for large datasets is very computationally intensive, there is a need to improve the performance using high performance computing techniques.

**Results:**

In this paper, Parallel Multicategory Support Vector Machines (PMC-SVM) have been developed based on the sequential minimum optimization-type decomposition method for support vector machines (SMO-SVM). It was implemented in parallel using MPI and C++ libraries and executed on both shared memory supercomputer and Linux cluster for multicategory classification of microarray data. PMC-SVM has been analyzed and evaluated using four microarray datasets with multiple diagnostic categories, such as different cancer types and normal tissue types.

**Conclusion:**

The experiments show that the PMC-SVM can significantly improve the performance of classification of microarray data without loss of accuracy, compared with previous work.

## Background

### Microarray data classification

In recent years the use of DNA microarrays has resulted in the creation of large datasets of molecular information characterizing complex biological systems. Machine learning algorithms applied to DNA microarray data based on molecular classification approach have shown to have statistical and clinical relevance for a variety of cancer types [1]. When applied to gene expression data, a classifier begins with a set of genes that have a common function. A separate set of genes that are known not to be members of the functional class is specified. These two sets of genes are combined to form a set of training examples in which the genes are labeled positively if they are in the functional class and are labeled negatively if they are known not to be in the functional class [2]. Using this training set, the classifier learns to discriminate between the members and non-members of a given functional class based on expression data. Having learned the expression features of the class, the classifier could recognize new genes as members or as non-members of the class based on their expression data. The classifier could also be reapplied to the training examples to identify outliers that may have previously been assigned to the incorrect class in the training set. Thus, the classifier would use the biological information in the investigator's training set to determine what expression features are characteristic of a given functional group and use this information to decide whether any given gene is likely to be a member of the group.

### Support Vector Machines

One strategy to solve classification problems is for the computer to learn the input/output functionality from training data. The learning algorithm attempts to find the underlying target function which maps from the input to the output. The estimate of the target function is known as the solution of the learning problem, which is chosen from a set of candidate functions that map from the input space to the output domain. These candidate functions are known as hypotheses. The learning algorithm takes the training data as input and selects a hypothesis from the hypotheses. The learning algorithm must have the ability to correctly classify data that are not in the training set, known as generalization [3].

When the learning problem deals with binary outputs, it is referred to as a binary classification problem, and when the problem deals with a finite number of outputs, it is referred as multi-category classification problem. In recent years, Support Vector machines (SVMs), a supervised machine learning algorithm is being used to solve many classification problems. SVM algorithm classifies the data by finding the optimal hyperplane between the classes of data. The focus is to maximise the margin between the parallel hyperplanes (to the optimal hyperplane). The training data which lie on the optimal hyperplane are called support vectors. This maximum-margin classifier was proposed by Vapnik Chervonenkis theory [4]. Many improvements have been developed in recent years for both binary and multi-category classification problems. The approaches based on [5-7] have produced impressive results. The algorithm based on [6], known as the sequential minimum optimization (SMO) algorithm [8,9], solves the multi-category problem indirectly as it breaks down the problem into independent binary classification problems. The approach in [7] solves the problem directly. The SMO algorithm can be parallelized as the smaller independent tasks can be distributed among the processing elements. The serial version of the SMO algorithm has been implemented and freely available at [10], named as LibSVM. The LibSVM code was used in this project to be parallelized. This algorithm is described in detail in a later section.

### Parallel classification

MC-SVMs generate a set of diagnostic models based on the training data and use decision functions to classify new datasets. Though the number of models generated is reduced by feature selection, the number of models generated is still very high [11]. This model generation is highly computationally intensive and time consuming. As a result there is a need to develop algorithms to reduce the execution time for the classification of gene expression data to make it more useful in practical applications. One approach is to decompose the large scale problem in the smaller problems and use multiple processors to solve the sub-problems concurrently and reduce the execution time. Unfortunately little work has been done in designing and developing parallel SVM algorithms. A parallel solver for large quadratic programs in training support vector machines was developed based on the SVM^*light *^[12] which is an implementation of Vapnik's Support Vector Machine [4]. However, this solver is developed for binary classification [13,14]. In our work, a new Parallel Multicategory Support Vector Machine (PMC-SVM) was developed based on SMO decomposition method for SVMs (SMO-SVM) and source code of libSVM. The SMO decomposition reduces the memory requirement of the algorithm and is an efficient implementation. The task of generating a large number of models is decomposed and distributed among multiple processing elements (PEs). The multicategory classification is broken down into smaller independent binary classification problems and assigned to each PEs. As a result the algorithm is very memory efficient. The PMC-SVM has been implemented in parallel using C++ and MPI, and tested on both shared-memory supercomputer and distributed-memory Linux cluster. The performance of PMC-SVM has been analyzed and evaluated based on four microarray datasets with multiple diagnostic categories [10,11]. The experiments show that the PMC-SVM can significantly improve the performance of classification without loss of accuracy, as compared with previous work.

## Results

### PMC-SVM implementation and software

The PMC-SVM was implemented using C++ and MPI based on the serial code available in LibSVM developed by Chih-Jen Lin based on SMO-SVM. The experiments were conducted on two platforms at the Mississippi Center for Super Computing Research (MCSR) located at the University of Mississippi [15]. One is the shared-memory SGI Origin 2800 Supercomputers (sweetgum) equipped with 128 CPUs, 64 gigabytes of memory, and 1.6 Terabytes of fiberchannel disk. The other is a distributed memory Linux cluster (mimosa) with 251 nodes. PBS (Portable Batch System) was used to submit the computational jobs to the two platforms. The PMC-SVM will become publicly available after further improvement and corroboration.

### Performance evaluation results

The parallel program was executed on mimosa and sweetgum separately using four different training datasets. The two datasets, Letter_scale and Mnist1_scale, are downloaded from [10]. The other two microarray datasets 14_Tumors and 11_Tumors are downloaded from [11]. The details of the four datasets are listed below

**Dataset 1**: Letter_scale

Classes: 26

Data size: 15,000 (training); 5,000 (testing)

Features: 16

**Dataset 2**: Mnist1_scale

Classes: 10

Data size: 21,000 (training), 49,000 (testing)

Features: 780

**Dataset 3**: 14_Tumors

Human tumor types: 14

Data file: 40 Mb

Normal tissue types: 12

**Dataset 4**: 11_Tumors

Data file: 18 Mb

Human tumor types: 11

To evaluate the performance of PMC-SVM, we have designed six computational experiments that are different combination of datasets and platforms, as listed in Table 1. The execution time and speedup corresponding to each experiment are given in Table 2. The speedup *S *is defined as



where *T*_*s *_is the serial execution time and *T*_*p *_the parallel execution time. For the convenience of comparison and analysis, the speedup and execution time are also plotted in Figures 2, 3, 4, 5 and 6.

### Performance improvement of PMC-SVM

From the experimental results, it is seen that the speedup increases when the number of processors is increased. Communication is needed only at the initial stage of parallel computing to dispatch the control parameters (e.g., the number of processing elements, p) and model parameters (e.g., the number of categories, *k*), and at the end of parallel model generation to collect all trained models. Thus, the speedup is close to ideal (linear) speedup and efficiency, defined as the ratio of the speedup to the number of PEs is high. In fact, when task decomposition technique, instead of data decomposition, is used, the communication cost is not significant in PMC-SVM. On both platforms, PMC-SVM significantly improves the performance of training multicategory models, compared with serial results, as shown in Figures 2, 3, 4 and 5.

### Prediction accuracy

After the PMC-SVM has been trained, the decision functions can be used to classify new microarray data. This part is not computationally intensive and can be easily implemented in parallel using data decomposition techniques. In case 1 with Letter-scale dataset, the training data size is 15,000 and testing data size 5,000. The classification accuracy of PMC-SVM is 82.24%. In case 2 with minist1_scale dataset, the training data size is 21,000 and testing data size is 49,000. The corresponding accuracy is 94.84%. The accuracy results cannot be directly compared with previous work [13] because of different implementation of SVM and techniques such as cross-validation used

## Discussion

### Influence of parallel platforms

PMC-SVM can be executed on both shared-memory supercomputer and a distributed Linux cluster without modifying the implementation when PBS is used to submit computation jobs. For the same dataset and the same number of processors, the execution time on sweetgum is less than that on mimosa (see Figure 3 and 5). This is because the sweetgum is a shared-memory platform on which the communication cost and the time to access the shared data is lower. For the distributed Linux cluster, it takes longer for two nodes to pass messages. While the execution times of PMC-SVM are different on two platforms, the speedups are very similar. The speedup on sweetgum is slightly higher than that on mimosa.

For a distributed memory system, each processor has a copy of data and communication cost is low, which is similar to the case with shared-memory system. However, each node needs the same amount of the memory to hold the entire dataset, which is memory inefficient. If data is also partitioned on a distributed memory system, it utilizes less memory but the communication cost is significant and hence affects the performance.

### Evaluation metric

To evaluate the performance of PMC-SVM, we may consider computation performance evaluation metric (speedup, memory and efficiency) and training/prediction accuracy. In this paper, we compare the results from serial and parallel programs. Both generate the same support vectors and give the same prediction accuracy, which verifies the correctness of the parallel design and implementation. Even if the same datasets were used by other researchers for training and testing SVM, it is hard to compare the results in this paper with previous work because the implementation and techniques in SVM are different. The paper focuses on the parallel design, implementation and the improvement of the computational performance, while prediction performance is part of the future work.

## Conclusion

PMC-SVM has been developed for classifying large datasets based on SMO-type decomposition method. For k category problem, the system generates *k(k-1)/2 *submodels, each model solves the corresponding subproblem, which is the most computationally expensive part in this algorithm. The computation task is partitioned and scheduled among all of the available processors to improve the performance. The PMC-SVM was implemented in MPI and C++ based on the serial implementation of SMO-SVM in libSVM. The performance is evaluated and analyzed on both shared-memory supercomputer (sweetgum) and distributed-memory Linux clusters (mimosa). As an application example, PMC-SVM was successfully trained using four microarray datasets for multicategory classification. The experimental results show that the high performance computing techniques and parallel implementation can achieve a significant speedup without loss of accuracy.

While this research focuses on classifying microarray data, the PMC-SVM system developed can be easily applied for multicategory classification of other large datasets.

## Methods

### Serial SMO-SVM

There are many implementations of SVM, such as libSVM and SVM^light^. libSVM was chosen as our basic serial program, which was implemented by Chih-Jen Lin based on SMO-SVM [10]. To elaborate the parallelization of PMC-SVM, we first briefly introduce SMO-SVM here.

The basic idea behind SVM is to separate two point classes of a training set,

*D *= {(*x*_*i*_,*y*_*i*_),*i *= 1,...*N*, *x*_*i *_∈ *R*^*n*^, *y*_*i *_∈ {-1,1}},     (2)

by using a decision function *F*:*R*^*n *^→ {-1,1} obtained by solving a convex quadratic programming optimization problem of the form

min *f*(α) = α ^*T*^*Q*α - *e*^*T*^α

Subject to 0≤α_*i*_≤*C*, *i *= 1,...,*l*,     (3)

*y*^*T*^α = 0

where *y *= [*y*_1_, *y*_2_, ..., *y*_*N*_]^*T*^, α = [α_1_, α_2_, ..., α_*N*_]^*T *^and *C *is a constant. *e *is a vector of all ones. *Q *is the symmetric positive semi-definite matrix and entries *Q*_*i, j *_are defined as

*Q*_*i*,*j *_= *y*_*i*_*y*_*j*_*K*(*x*_*i*_,*x*_*j*_), *i, j *= 1,2 ..., *N*,     (4)

where *K*(·, ·) denotes a kernel function, such as linear kernel, polynomial kernel and radial basis function (RBF). RBF kernel is used in the numerical experiments of this project.

Currently, decomposition method is one of the major methods to train SVM, in which only a subset of variable is considered per iteration. The subset, denoted as B, is called working set. If B is restricted to have only two elements, this special type of decomposition method is the Sequential Minimal Optimization (SMO). There are four steps to implement SMO [8]:

1. Find α^1 ^as the initial feasible solution. Set *k *= 1.

2. If α^*k*^is a stationary point of (2), stop. Otherwise, find a two-element working set *B *= {*i*,*j*}⊂{1,..., l}. Define *N *≡ {1,..., l}\ *B*, and  and  as sub-vector of α^*k*^corresponding to B and N, respectively.

3. If *a*_*i*,*j *_= *K*_*ii *_+ *K*_*jj *_- 2*Kij *> 0

Solve the following sub-problem with the variableα_*B *_:



else

Solve



*subject to constra *int *s of *(5)

4. Set  to be the optimal solution of (4) and  Set *k *← *k *+ 1 and go to step 2.

After the PMC-SVM is trained, the decision functions are used to classify new datasets.

### Parallelization

#### Task decomposition

**Figure 1:**
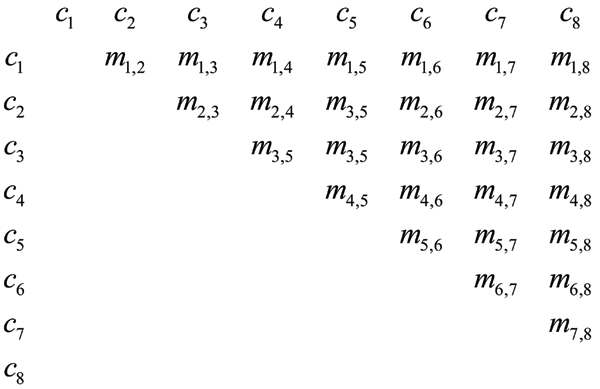
Categories (*C*_*i*_) and submodels (*m*_*i*, *j*_) in PMC-SVM.

**Figure 2:**
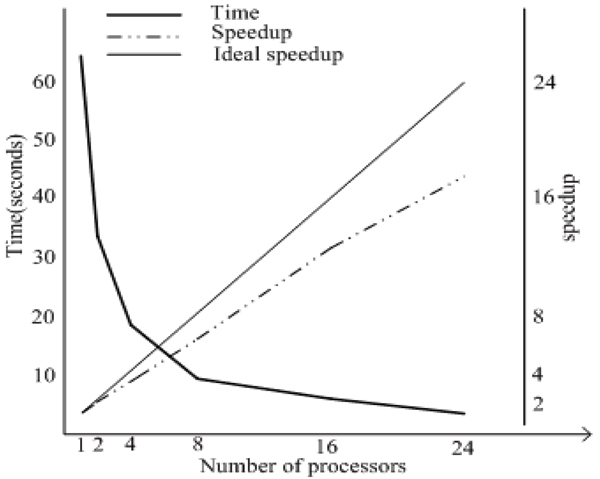
The execution time and speedup of PMC-SVM on sweetgum with Dataset 1 (Letter_scale).

**Figure 3:**
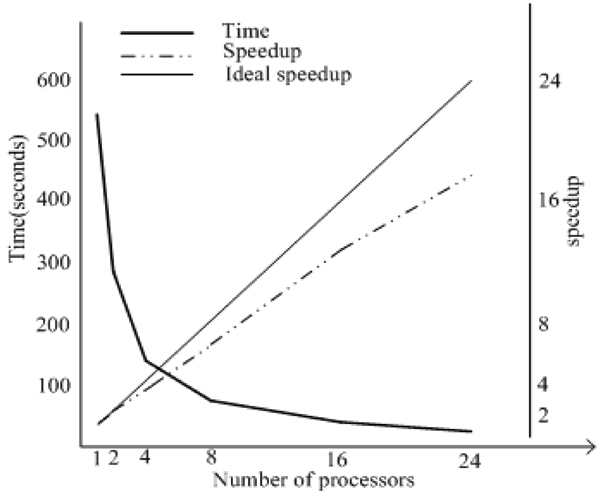
The execution time and speedup of PMC-SVM on sweetgum with Dataset 2 (Mnist1_scale).

**Figure 4:**
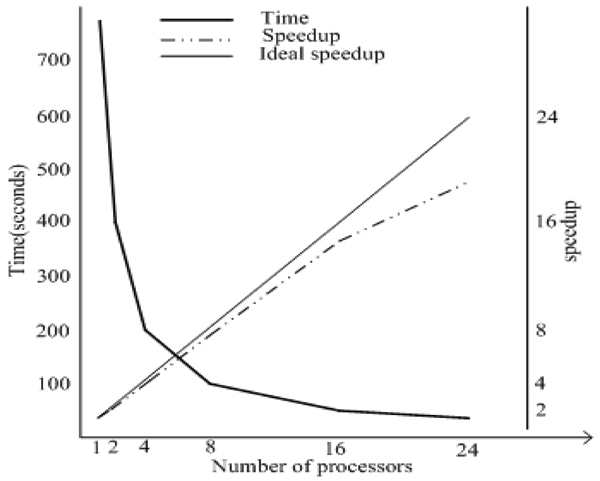
The execution time and speedup of PMC-SVM on sweetgum with Dataset 3 (14_Tumors).

**Figure 5:**
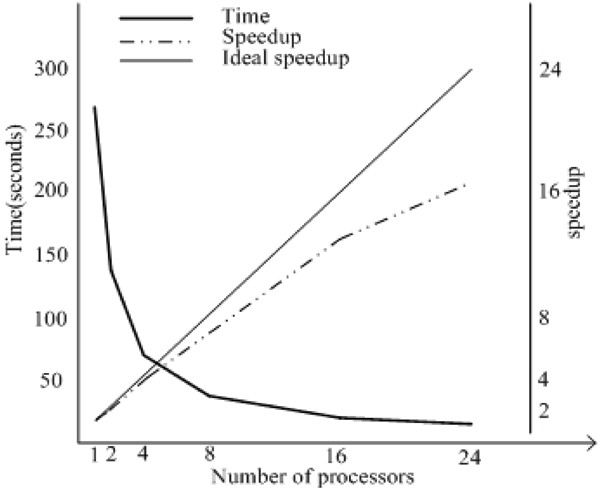
The execution time and speedup on sweetgum with Dataset 4 (11_Tumors).

**Figure 6:**
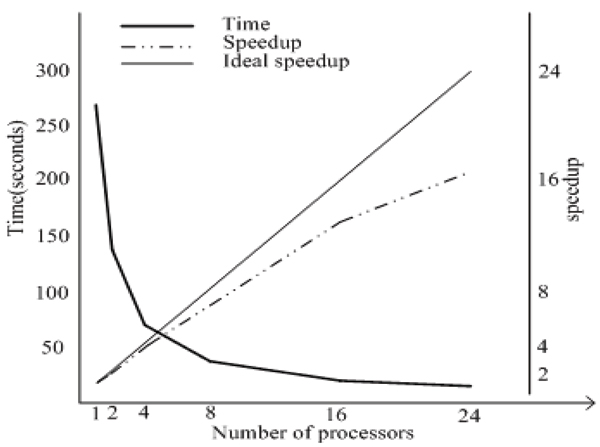
The execution time and speedup of PMC-SVM on mimosa with Dataset 2 (Mnist1_scale).

Task decomposition is the first step for the parallel algorithm design. In multicategory classification of support vector machines, the algorithm will generate multiple binary SVM models, denoted by *m*_*i, j*_, *i, j *= 1,...,*k*, for *k *categories, each denoted by *C*_*i*_, *i *= 1,...,*k*. Each model *m*_*i, j *_is generated from categories *C*_*i *_and *C*_*j*_. A total of  submodels are generated from k categories in MC-SVM. For example, if we have 4 processors and 8 categories, 28 models will be generated, as shown in Figure 1. Generating submodels is the most time consuming task in MC-SVM so it is efficient to distribute the task onto multiple processors and each processor can perform different subtasks concurrently. To achieve the optimal performance, execution time, communication cost among processors, task dependency and memory utilization are analysed. From figure 1, it is intuitive to decompose the datasets so that each PE generates a number of submodels based on a subset of training data. This decomposition technique is memory efficient but it results in large communication cost because one PE needs to receive certain category of data from other PEs in order to generate a submodel. The communication cost can affect the performance of this parallel model. It is observed that training each submodel only needs two categories of data and all submodels are independent. Thus, an alternative approach is to partition the task of generating submodels and to dispatch the subtasks among multiprocessors. For example, the 28 submodels in Figure 1 can be distributed among 4 PEs and each PE performs the subtask of generating 7 submodels. This technique can achieve better load balance. Task scheduling is described in the next section.

#### Task scheduling algorithm

When *k*_0 _submodels are assigned to p PEs, each PE performs the generation task of  submodels. A task scheduling algorithm is needed to determine which categories of data are used by each PE. The pseudocode of scheduling tasks is given as follows

Task scheduling algorithm

// Task scheduling algorithm for PMC-SVM training

// model_no: the index of models

// p: the total number of PEs.

// my_rank: processor index, 0<=rank <p

// i, j: class index

// k: total number of classes

model_no = 0;

for ( int i = 2; i <= k; i++){

for( int j = 1; j < i; j++){

model_no++;

if(my_rank = = model_no % p);

// processor with my_rank gets class i and class j

get_data(i) // read class i data

get_data(j) // read class i data

generate_bin_svm(i,j); // generate a submodel for category i and j

}

}

With this scheduling algorithm, tasks are evenly distributed among multiple processors and achieve better balance.

### Parallel job submission

The Portable Batch System (PBS) is a flexible workload management system [15]. It provides users with a single coherent interface to all their computing resources. It can improve understanding of computational requirements and user needs and provides a more cost-effective solution. The PBS used to submit a job to sweetgum machine is given below

#PBS -S /bin/tcsh

# Set up the cpus and memory

#PBS -l ncpus = 4

# Name this job "job_svm"

#PBS -N job_svm

rm job_svm.o* # Remove any PBS output files from previous runs

rm job_svm.e* # Remove any PBS error files from previous runs

# Do our job

# make the makefile

make

# run the svm-train, save the file to svm8.out

/usr/local/appl/mpich-1.2.1/bin/mpirun -np 8 svm-train letter.scale >> svm.out

## Authors' contributions

CZ and YD initiated the project and carry out the design and analysis. PL and AR implemented the PMC-SVM and evaluated the performance. All authors contributed and approved the final manuscript.

## Figures and Tables

**Table 1:** Sample Testing cases of PMC-SVM

**Experiments**	**Datasets**	**Platforms**	**Results**
Case 1	Dataset 1 Letter_scale	sweetgum (SGI 2800)	Table 2 Figure 2
Case 2	Dataset 2 Mnist1_scale	sweetgum (SGI 2800)	Table 2 Figure 3
Case 3	Dataset 3 14_Tumors	sweetgum (SGI 2800)	Table 2 Figure 4
Case 4	Dataset 4 11_Tumors	sweetgum (SGI 2800)	Table 2 Figure 5
Case 5	Dataset 2 Mnist1_scale	mimosa (Linux cluster)	Table 2 Figure 6

**Table 2:** Execution time T(s) and Speedup S for different cases

# of PEs	Case 1		Case 2		Case 3		Case 4		Case 5	
	
	T	S	T	S	T	S	T	S	T	S
1	64.23	-	549.62	-	592.13	-	774.29	-	260.13	-
2	33.26	1.93	278.49	1.97	299.85	1.97	390.13	1.98	132.17	1.96
4	17.17	3.74	142.35	3.86	153.47	3.85	197.56	3.92	68.09	3.82
8	9.53	6.73	75.08	7.32	79.10	7.49	100.73	7.69	36.72	7.08
16	5.19	12.37	41.25	13.32	44.26	13.38	52.56	14.73	20.25	12.85
24	3.60	17.84	31.10	17.67	35.79	16.54	42.52	18.21	15.91	16.35

## References

[B1] Mukherjee S, Tamayo P, Mesirov JP, Slonim D, Verri A, Poggio T (1999). Support Vector Machine Classification of Microarray Data. CBCL Paper #182/AI Memo #1677.

[B2] Brown MPS, Grundy WN, Lin D, Cristianini N, Sugnet C, Furey TS, Ares M, Haussler D Knowledge-based analysis of microarray gene expression data using support vector machines. Proc Natl Acad Sci.

[B3] Cristianni Nello, Shawe-Taylor John (2000). An Introduction to Support Vector Machines.

[B4] Vladimir N, Vapnik (1995). The Nature of Statistical Learning Theory.

[B5] Weston J, Watkins C Support vector machines for multi-class pattern recognition. Proceedings of the Seventh European Symposium On Artificial Neural Networks (ESANN 99).

[B6] Platt JC (1998). Fast Training of support vector machines using sequential minimal optimization. Advances in Kernel Meothds – Support Vector Learning.

[B7] Crammer Koby, Singer Yoram (2001). On the Algorithmic Implementation of Multiclass Kernel-based Vector Machines.

[B8] Chen Pai-Hsuen, en Fan Rong, Lin Chih-Jen (2005). A study on SMO-type decomposition methods for support vector machines. Technical report.

[B9] Hsu Chih-Wei, Lin Chih-Jen (2002). A comparison of methods for multi-class support vector machines. IEEE Transactions on Neural Networks.

[B10] Chang Chih Chung, Lin Chih Jen (2005). LIBSVM: a library for support vector machines. http://www.csie.ntu.edu.tw/~cjlin/libsvmtools/datasets/.

[B11] Statnikov Alexander, Aliferis ConstantinF, Tsamardinos Loannis, Hardin Douglas, Levy Shawn (2005). A comprehensive evaluation of multicategory classification methods for microarrary genen expression cancer diagnosis. Bioinformatics.

[B12] SVM^*light *^Support Vector Machine. http://www.cs.cornell.edu/People/tj/svm_light/.

[B13] Zanghirati G, Zanni L (2003). A Parallel Solver for Large Quadratic Programs in Training Support Vector Machines. Parallel Computing.

[B14] Zanni L, Serafini T, Zanghirati G (2005). A Parallel Software for Training Large-Scale Support Vector Machines on Multiprocessor Systems. Technical Report 71.

[B15] Mississippi Center for Supercomputing Research(MCSR). http://www.mcsr.olemiss.edu/.

